# A novel glucagon analog with an extended half-life, HM15136, normalizes glucose levels in rodent models of congenital hyperinsulinism

**DOI:** 10.1038/s41598-022-21251-y

**Published:** 2022-10-06

**Authors:** Yong Ho Heo, Jung Kuk Kim, Jong Suk Lee, Sang-Hyun Lee, Seung-Hyun Shin, In Young Choi, Ha Hyung Kim

**Affiliations:** 1 Hanmi Research Center, Hanmi Pharmaceutical Co. Ltd., 550 Dongtangiheung-ro, Hwaseong-si, 18469 Gyeonggi-do Republic of Korea; 2 Biotherapeutics and Glycomics Laboratory, College of Pharmacy, Chung-Ang University, 84 Heukseok-ro, Dongjak-gu, Seoul, 06974 Republic of Korea; 3 Department of Global Innovative Drugs, Graduate School of Chung-Ang University, 84 Heukseok-ro, Dongjak-gu, Seoul, 06974 Republic of Korea

**Keywords:** Molecular medicine, Pharmacology

## Abstract

Congenital hyperinsulinism (CHI) is a rare genetic condition characterized by uncontrolled insulin secretion, resulting in hypoglycemia. Although glucagon has lately been regarded as a therapeutic option for CHI, its use is severely hampered by its poor solubility and stability at physiological pH, as well as its short duration of action. To address these constraints, we developed HM15136, a novel long-acting glucagon analog composed of a glucagon analog conjugated to the Fc fragment of human immunoglobulin G4 via a polyethylene glycol linker. In this study, we established that HM15136 was more soluble than natural glucagon (≥ 150 mg/mL vs 0.03 mg/mL). Next, we confirmed that HM15136 activated glucagon receptor in vitro and induced glycogenolysis and gluconeogenesis in rat primary hepatocytes. Pharmacokinetics (PK)/Pharmacodynamics (PD) analysis of HM15136 shows that HM15136 has a markedly longer half-life (36 h vs. < 5 min) and increased bioavailability (90%) compared to native glucagon in mice. Further, HM15136 could effectively reverse acute hypoglycemia induced by insulin challenge, and multiple doses of HM15136 could sustain increased blood glucose levels in CHI rats. In conclusion, our findings indicate that HM15136 promotes sustained elevation of blood glucose, demonstrating the potential for development as a once-weekly therapy for CHI.

## Introduction

Congenital hyperinsulinism (CHI) is a group of genetic disorders characterized by the impaired regulation of insulin production in response to blood glucose (BG) levels, resulting in excess insulin. Affected individuals experience recurrent episodes of hyperinsulinemic hypoglycemia due to inappropriate elevation of serum insulin^1–5^. The pathogenesis of CHI is distinct from that of insulin-related hypoglycemia due to the insulin resistance syndromes as well as acquired hyperinsulinemic hypoglycemia. In CHI, abnormalities in the insulin secretory mechanism or glucose-sensing mechanism result in a failure to regulate pancreatic insulin secretion in response to hypoglycemic conditions (serum glucose level < 60 mg/dL). This leads to high circulating insulin levels that promote hepatic and skeletal muscle glycogenesis, causing a decrease in the amount of free glucose available in the bloodstream and the suppression of free fatty acid (FFA) formation, an alternative energy substrate for the brain^6^.

The global estimate of CHI prevalence is 1 in 50,000^7^. If left untreated, severe hypoglycemia due to CHI can lead to secondary brain damage or death. Although it was initially thought to affect only infants and children, several cases have been reported in adults of all ages, albeit at a much lower incidence^8^. In the UK, it is predicted that approximately 95 neonates are born with the disease each year^7^. CHI can be categorized into two subtypes—focal disease and diffuse disease. The focal disease subtype refers to CHI caused by an abnormality in a discrete region of the endocrine pancreas. In contrast, the diffuse subtype of CHI is caused by dysregulated insulin production in all pancreatic beta cells^9^.

Treatment of CHI focuses on restoring the decreased plasma glucose levels to the normal range and reducing insulin levels to prevent neurological complications. Dextrose infusions have been applied to increase BG levels in patients with CHI^10^. However, oral dextrose does not effectively prevent severe hypoglycemia, and high concentrations of dextrose require intravenous infusion, resulting in limited efficacy^11^. Glucagon inhibits glycogenesis and promotes glycogenolysis and gluconeogenesis, increasing BG levels. Studies have been conducted on dose-titrating regimens of glucagon infusion to achieve glycemic stability and avoid hypoglycemia in CHI patients, suggesting the potential of glucagon infusion as a therapeutic option for severe hypoglycemia in CHI patients^10,12,13^. However, existing formulations of glucagon show poor solubility and stability. Therefore, there is an urgent need to develop a long-lasting glucagon analog that is active under physiological conditions.

Treatment options other than glucagon have been investigated for the treatment of CHI. Diazoxide, a first-line therapy, is a nondiuretic benzothiadizine originally formulated as a peripheral vasodilator for the treatment of hypertension. A meta-analysis of six cohorts (1142 subjects) reported that the response rate of diazoxide therapy for hyperinsulinemic hypoglycemia was 71%; however, it also caused some adverse effects (e.g., hypertrichosis)^14^. Octreotide—a synthetic peptide analog of somatostatin—has been frequently used as a second-line therapy to treat patients with CHI who are not responsive to diazoxide. However, the somatostatin peptide analog has other effects, including suppression of luteinizing hormone to gonadotropin-releasing hormone, a decrease in splanchnic blood flow, and inhibition of the release of vasoactive intestinal peptide, secretin, motilin, serotonin, gastrin, and pancreatic polypeptide^15^. Patients not responsive to octreotide and diazoxide require surgical resection of the pancreas, physically removing ~ 95% of insulin-secreting cells to attenuate their severe hypoglycemia. However, most patients who have undergone pancreatectomy, present with postoperative complications such as recurrent hyperinsulinemic hypoglycemia and adverse effects such as diabetes mellitus and exocrine pancreatic insufficiency^16,17^. Additionally, to reduce insulin levels, mTOR inhibitors have been investigated for the treatment of hyperinsulinemic hypoglycemia (e.g., CHI). However, mTOR inhibitors caused many adverse effects, including immunosuppression, stomatitis, decreased kidney function, and pneumonitis^16^.

In this study, we have developed HM15136 by conjugating the Fc fragment of IgG4 to GC15136 via a polyethylene glycol (PEG) linker to enhance stability under physiological conditions. Our results from in vitro and in vivo experiments demonstrate that HM15136 is more stable than native glucagon. We confirmed that HM15136 increases BG in the Sprague–Dawley (SD) rat model and shows long-lasting PK properties in mice, rats, and dogs. Collectively, our results suggest that HM15136, showing higher solubility and stability than native glucagon, could be a good candidate for the treatment of CHI.

## Results

### Glucagon analog was modified to enhance solubility by conjugating GC15136 and HMC001

**Figure 1:**
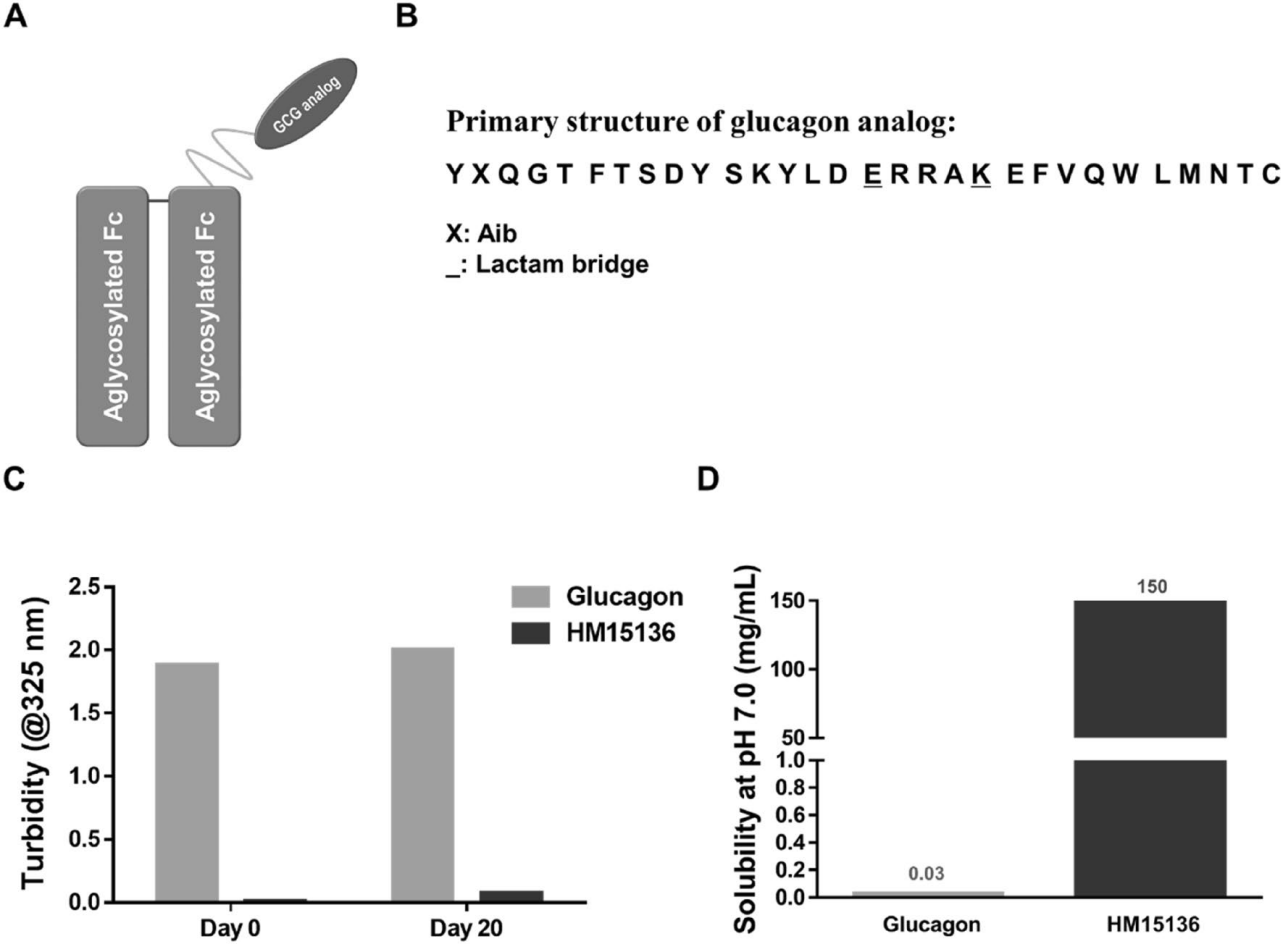
Structure of HM15136, conjugate of glucagon analog and human immunoglobulin G4 Fc. (**A**) Structure of HM15136. (**B**) Primary sequence of glucagon analog. (**C**) Physical stability of HM15136 in phosphate-buffered saline (pH 7.0) at 25 °C. (**D**) Solubility of HM15136 (pH 7.0) at 25 °C.

To develop a glucagon analog with enhanced stability and long-lasting effects, we attached a linker between a glucagon analog (GC15136) and the Fc fragment of human immunoglobulin G4 (HMC001). GC15136 and HMC001 are linked via a 10-kDa, bifunctional maleimide-polyethylene glycol-aldehyde (MAL-PEG-ALD) linker (Fig. 1A). GC15136 is a chemically synthesized glucagon analog (Fig. 1B). The conjugation of GC15136 and HMC001 is carried out through the formation of a thioether and amine bond between the hetero-bifunctional MAL-PEG-ALD, Cys30 in GC15136, and the N-terminal amino acid in HMC001. The molecular weight of HM15136 is approximately 63 kDa for the whole molecule and 53 kDa for the protein content. The scattering of incident light at 325 nm was measured as an indicator of solubility in phosphate-buffered saline (PBS, pH 7.0) at two different time points. Compared to native glucagon, HM15136 showed markedly lower turbidity at both days 0 and 20 after addition to PBS, indicating that the solubility of HM15136 is much higher than that of the native glucagon (Fig. 1C). Thus, we demonstrated that HM15136 shows dramatically increased solubility at pH 7.0 compared to native glucagon (≥ 150 mg/mL vs. 0.03 mg/mL) (Fig. 1D).

### HM15136 controls glucose homeostasis through the same mechanism as glucagon

The glucagon analog (GC15136) of HM15136 is responsible for increasing BG levels for the treatment of CHI. Glucagon acts through the glucagon receptor (GCGR), a member of the G protein-coupled receptor family, predominantly expressed in the liver^18^. Upon GCGR stimulation, glucagon activates adenylate cyclase to generate intracellular cAMP^19^. The increased cAMP levels activate glycogen phosphorylase and fructose-2,6-bisphosphatase, stimulating glycogenolysis and gluconeogenesis, respectively, leading to increased hepatic glucose production^20^.

To investigate the biological functions of HM15136, cAMP accumulation in response to HM15136 was examined using hGCGR/CHO cells in which the endogenous rodent glucagon receptor was replaced with the human glucagon receptor (hGCGR). These cells accumulate cAMP in response to native glucagon or glucagon analogs.

**Figure 2:**
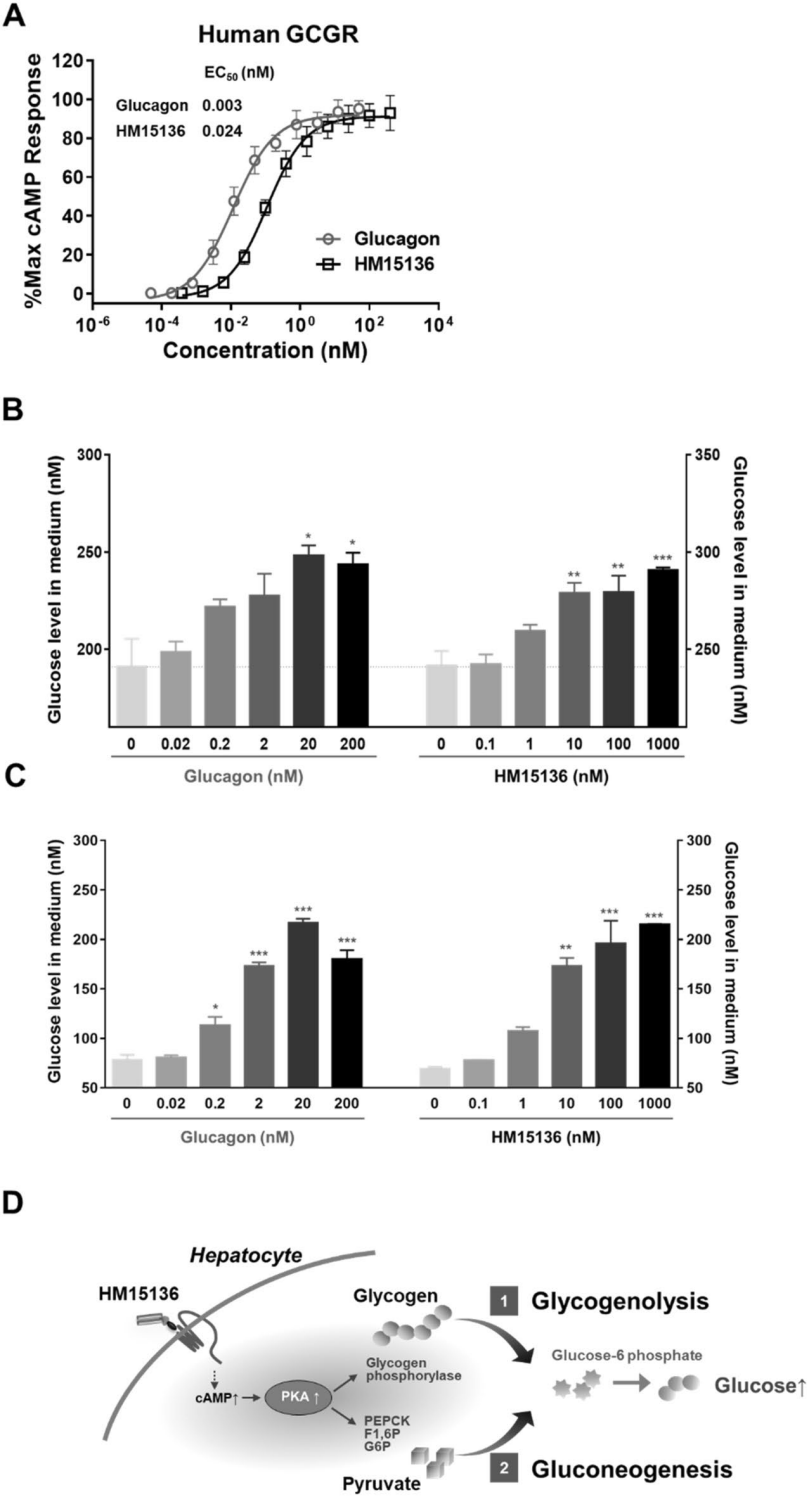
HM15136 stimulates GCCR and increases cAMP levels. (**A**) HM15136 induced a dose-dependent increase in intracellular cAMP levels in hGCGR/CHO cells. Symbols denote native glucagon (open circle) and HM15136 (open square). The concentration of individual test agents ranged from 0.0002 to 194.4 nM (for native glucagon) and from 0.0015 to 1555.2 nM (for HM15136). (**B,C**) Glucose production in rat primary hepatocyte treated with either HM15136 or native glucagon for 30 min (**B**, for glycogenolysis) and 6 h (**C**, for gluconeogenesis). Isolated hepatocytes were incubated with insulin to promote glycogen production and then, treated with HM15136 for 30 min to induce glycogenolysis. For gluconeogenesis, serum-starved hepatocytes were treated with HM15136 for 6 h in the presence of pyruvate and lactate. After treatment, the glucose level in the collected medium was determined via a GOPOD assay. Results represent mean ± S.D. of duplicate assessments. *p < 0.05, **p < 0.01, ***p < 0.001 vs 0 nM. (**D**) Mechanism of HM15136 for elevation of glucose blood level.

HM15136 induced intracellular cAMP generation in a dose-dependent manner and with a full-agonistic, maximal efficacy similar to that of native glucagon. The EC_50_ of HM15136 was 0.024 ± 0.006 nM while that of native glucagon was 0.003 ± 0.001 nM for hGCGR. Based on these observed EC_50_ values, HM15136 is 12.7 ± 5.5% as potent as glucagon at activating hGCGR (Fig. 2A).

Stimulation of the GCGR results in the production and release of glucose via glycogenolysis and gluconeogenesis in the liver^21^. Of note, while BG levels are preferentially maintained by glycogenolysis—the breakdown of stored glycogen during the phase of early fasting, gluconeogenesis—glucose production using non-carbohydrate carbon substrates—becomes the most prominent route of BG maintenance after glycogen depletion under conditions of prolonged starvation^22^. Therefore, we examined the short- and long-term glucose-producing potential of HM15136 by evaluating glycogenolysis and gluconeogenesis in rat primary hepatocytes, respectively.

Similar to native glucagon, HM15136 induced glucose production via both glycogenolysis and gluconeogenesis in rat primary hepatocytes, suggesting that HM15136 possesses bona fide glucagon-like pharmacological activity. The effective concentration of HM15136 was 1–1000 nM, compared to 0.2–200 nM for native glucagon, suggesting that its relative potency was approximately 20% that of native glucagon (Fig. 2B–D). Conjugation of HMC001 to GC15136 appeared to decrease the intrinsic in vitro activity of HM15136 probably via its steric hindrance effect, which could be compensated for by the prolonged duration of action of HM15136 in vivo related to its improved PK properties.

### HM15136 binds to FcRn in a pH-dependent manner and does not interact with FcγR or C1q

The pH-dependent binding of IgGs to FcRn plays a critical role in maintaining the in vivo circulation of IgGs. The interaction between Fc and FcRn is strictly pH-dependent that strong interactions between IgGs and FcRn occur under acidic pH conditions (pH 6.0). However, the binding affinity decreases considerably under neutral/physiological pH conditions (pH 7.4). This interaction between IgG and FcRn is responsible for protecting IgG from lysosomal degradation, which may be related to the decreased vascular clearance of IgG^23^. This property of IgGs has been exploited to prolong the duration of action of HM15136 in vivo.

Binding to FcRn was evaluated using surface plasmon resonance (SPR) technology. FcRn are widely expressed in endothelial and epithelial cells^24^. HM15136 demonstrated concentration-dependent association with FcRn at pH 6.0 (K_a_ = 4.2 × 10^5^), similar to HMC001 (K_a_ = 8.5 × 10^5^). Both HM15136 and HMC001 rapidly dissociated from FcRn at pH 7.4, demonstrating the typical, pH-dependent, FcRn-binding property (Supplementary Fig. 1A,B). These data predict that HM15136 will exhibit an extended duration of action in vivo.

We expected minimal effector functions (e.g., antibody-dependent cellular cytotoxicity and complement-dependent cytotoxicity) of the HMC001 moiety in HM15136 as it is derived from the constant region of human IgG4^25^. Furthermore, because HMC001 is derived from *Escherichia coli* and lacks glycosylation, HM15136 is unable to bind FcR or C1q and, thus, is not expected to exert any effector functions.

**Figure 3:**
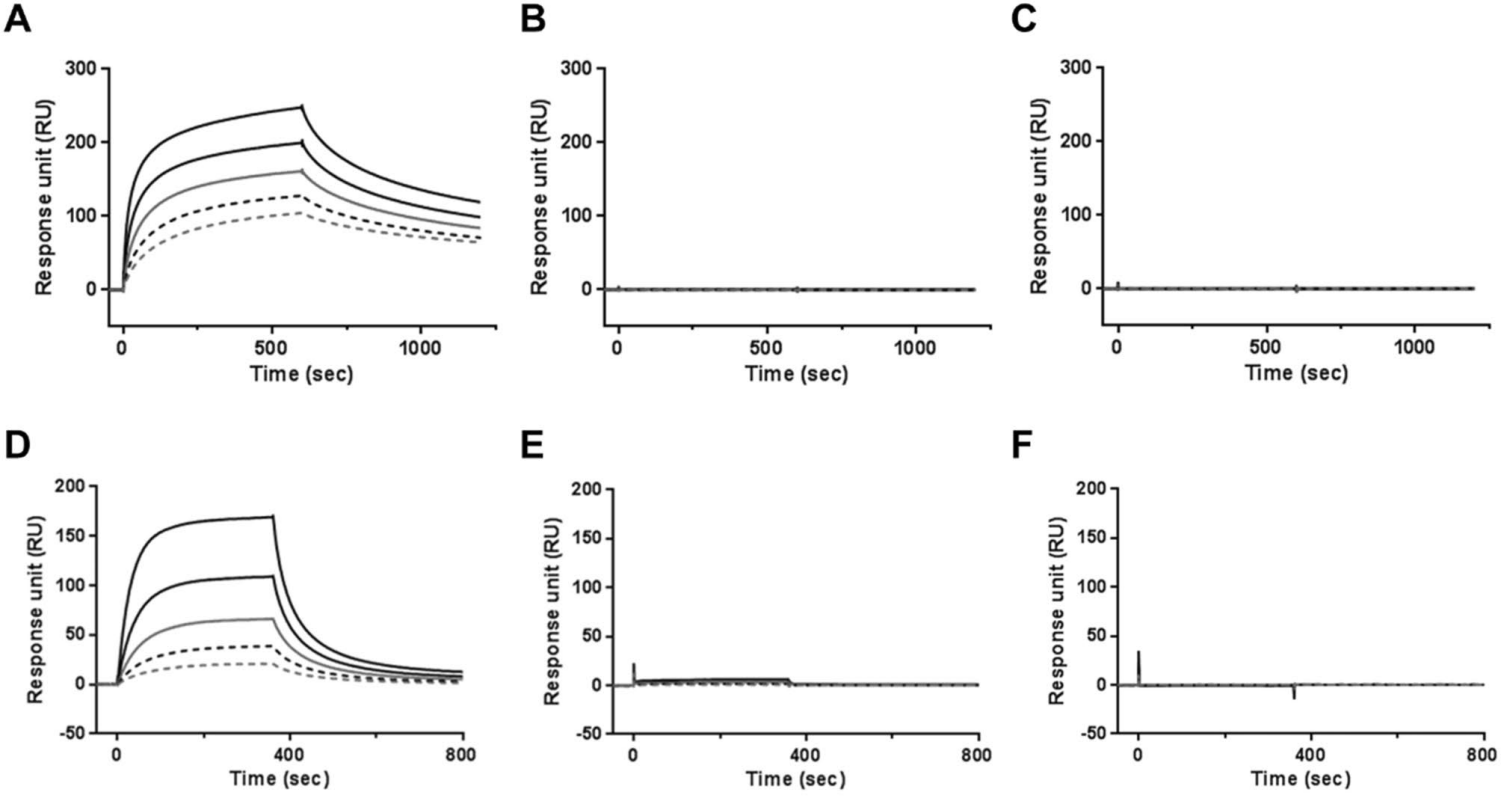
HM15136 interacts with FcRn and does not bind FcγR. The sensorgrams for the FcγRIA (**A–C**), C1q (**D–F**) binding of IgG1 (= I.V.-Globulin SN), HMC001, and HM15136. IgG1 control (**A,D**), HMC001 (**B,E**) and HM15136 (**C,F**). The concentration of individual test from top to bottom were 500 nM, 250 nM, 125 nM, 62.5 nM, and 31.25 nM for IV-Globulin SN (**A**) and HMC001 (**B**) and 486 nM, 243 nM, 121.5 nM, 60.75 nM, and 30.38 nM for HM15136 (**C**). The concentration of the individual test agents: IV-Globulin SN (**D**), HMC001 (**E**), and HM15136 (**F**) from top to bottom were 50 nM, 25 nM, 12.5 nM, 6.25 nM, 3.125 nM for C1q.

To confirm that the effector function is completely absent in HM15136, the binding properties of HM15136 and HMC001 to commercially available FcγR subtypes (FcγRIA, FcγRIIA, FcγRIIB/C, FcγRIIIA, and FcγRIIIB) and human C1q, the first subcomponent of the C1 complex of the classical pathway of complement activation, were evaluated using SPR analysis. The results clearly indicated that neither HM15136 nor HMC001 bind to FcγRs or C1q. In contrast, the positive control IgG1^26^ demonstrated robust binding in this model system (Fig. 3A–F, Supplementary Table 1). Taken together, these data suggest that HM15136 should not exhibit any effector functions via binding to FcγR or C1q in vivo*.*

### HM15136 shows long-lasting PK properties in preclinical models

**Figure 4:**
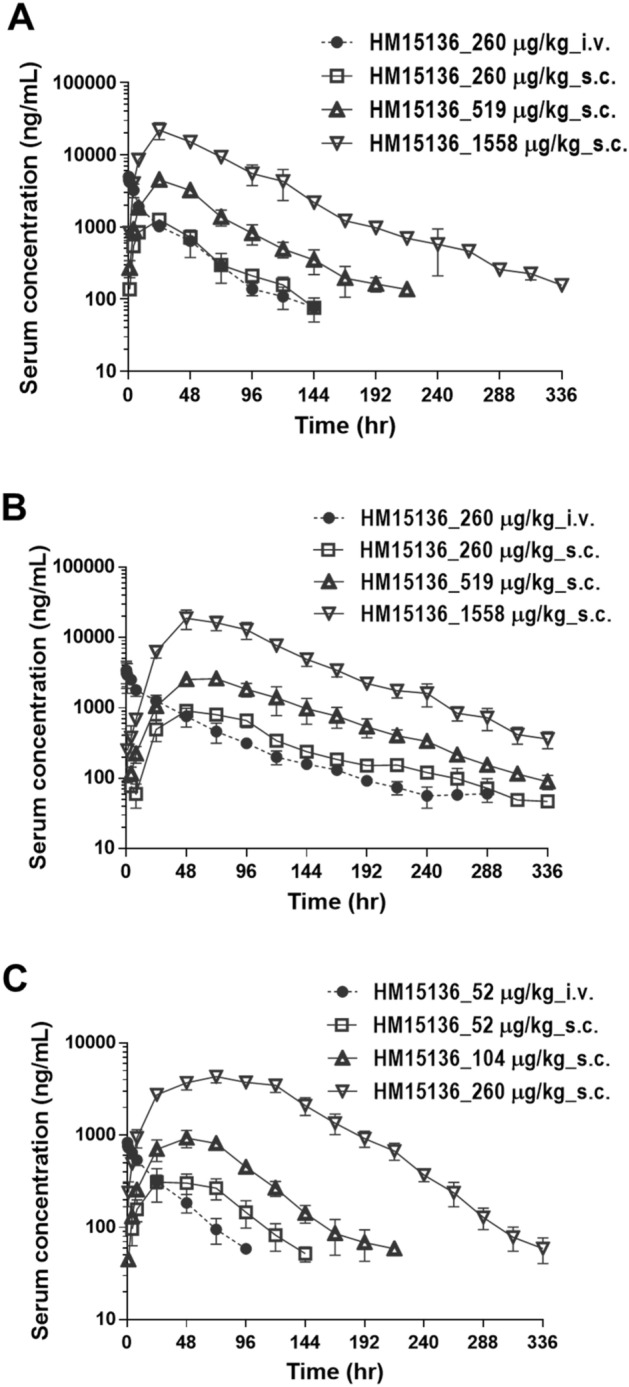
HM15136 exhibited good bioavailability and had high systemic exposure and long-lasting PK properties in mice, rat, and dog. (**A**) A single dose of HM15136 was administered via the intravenous and subcutaneous routes in ICR mice (n = 3/time point/group). The error bars indicate standard deviation. The symbols represent the following: (filled circle) HM15136 (i.v.) 260 μg/kg; (open square) HM15136 (s.c.) 260 μg/kg; (open triangle) HM15136 (s.c.) 519 μg/kg; (open reverted triangle) HM15136 (s.c.) 1558 μg/kg; (**B**) A single dose of HM15136 was administered via the intravenous and subcutaneous routes in SD rats (n = 3/group). The error bars indicate standard deviation. The symbols represent the following: (filled circle) HM15136 (i.v.) 260 μg/kg; (open square) HM15136 (s.c.) 260 μg/kg; (open triangle) HM15136 (s.c.) 519 μg/kg; (open reverted triangle) HM15136 (s.c.) 1558 μg/kg. (**C**) A single dose of HM15136 was administered via the intravenous and subcutaneous routes in beagle dogs (n = 3/group). The error bars indicate standard deviation. The symbols represent the following: (filled circle) HM15136 (i.v.) 52 μg/kg; (open square) HM15136 (s.c.) 52 μg/kg; (open triangle) HM15136 (s.c.) 104 μg/kg; (open reverted triangle) HM15136 (s.c.) 260 μg/kg.

PK studies of HM15136 were conducted using mice, rats, and dogs and are summarized in Table 1. Serum concentrations of HM15136 were determined using an enzyme-linked immunosorbent assay (ELISA). PK parameters were calculated by non-compartmental analysis using PhoenixTM WinNonlin® 8.0 (Pharsight, USA). The mean concentration versus time profiles of HM15136, and the corresponding PK parameters are summarized (Fig. 4 and Table 1).

**Table 1:** Summary of HM15136 PK parameters in animals.

Species (no. of animals/sex)	Mouse (n = 3/time point/group, male)		Rat (n = 3/group, male)		Dog (n = 3/group, male)	
Route	IV	SC	IV	SC	IV	SC
Dose^a^ (μg/kg)	260	260	260	260	52	52
		519		519		104
		1558		1558		260
C_0_ or C_max_ (ng/mL)	5495.1	1263.9	3757.0 ± 1196.0	905.7 ± 136.0	901.9 ± 72.1	325.6 ± 104.0
		4508.3		2664.3 ± 362.6		939.9 ± 186.5
		22,091.1		18,938.4 ± 5627.8		4360.6 ± 612.4
T_max_ (h)	–	24.0	–	48.0 ± 0.0	–	32.0 ± 13.9
		24.0		64.0 ± 13.9		56.0 ± 13.9
		24.0		56.0 ± 13.9		64.0 ± 13.9
t_1/2_ (h)	54.5	32.3	66.4 ± 33.0	40.9 ± 17.7	24.9 ± 2.2	26.6 ± 1.5
		56.2		46.4 ± 1.8		32.6 ± 12.9
		53.8		54.8 ± 8.7		34.9 ± 3.7
AUC_last_ (ng·h/mL)	89,253.7	68,605.9	112,670.4 ± 22,228.3	100,461.1 ± 12,112.2	22,309.9 ± 4093.9	27,271.2 ± 8067.6
		268,111.5		307,765.0 ± 67,995.6		81,676.7 ± 6672.7
		1,509,634.6		1,842,306.4 ± 378,363.3		570,046.2 ± 65,377.0
AUC_INF_ (ng·h/mL)	95,318.7	72,167.4	117,802.7 ± 24,433.7	103,325.8 ± 10,975.4	24,466.9 ± 4218.0	29,278.0 ± 8370.0
		279,211.6		313,769.7 ± 68,976.0		84,208.4 ± 7085.7
		1,521,803.5		1,870,651.6 ± 383,712.6		573,029.6 ± 65,265.7
MRT_last_ (h)	31.0	45.6	54.8 ± 5.4	103.4 ± 4.9	27.5 ± 4.4	57.9 ± 6.2
		55.7		106.3 ± 1.2		69.4 ± 7.5
		67.4		97.4 ± 1.8		100.2 ± 6.4
CL or CL/F (mL/h/kg)	2.7	3.6	2.3 ± 0.5	2.5 ± 0.3	2.2 ± 0.4	1.9 ± 0.5
		1.9		1.7 ± 0.4		1.2 ± 0.1
		1.0		0.9 ± 0.2		0.5 ± 0.1
V_d_ or V_d_/F (mL.kg)	214.6	167.9	207.1 ± 74.9	154.0 ± 83.9	77.3 ± 7.6	71.1 ± 15.3
		150.7		114.0 ± 22.8		57.9 ± 20.8
		79.4		68.6 ± 23.2		22.9 ± 1.8
Bioavailability (%)^b^	–	76.9	–	89.2	–	> 100
		> 100		> 100		> 100
		> 100		> 100		> 100
Study no.	Kv-7031		Kv-7025		Kv-7032	

*IV* intravenous, *M*  male, *SC*  subcutaneous, *CL*  clearance, *F*  bioavailability.

^a^Vehicle: 20 mM Na-citrate (pH 5.0), 0.1 mg/mL l-methionine, 8% sucrose, 0.02% polysorbate 20.

^b^Bioavailability was calculated based on AUC^last^.

Mouse PK properties of HM15136 were evaluated after a single intravenous (IV) administration at a dose of 260 μg/kg and following subcutaneous (SC) administration at doses of 260 μg/kg, 519 μg/kg, and 1558 μg/kg in male mice (Institute for Cancer Research [ICR] mice) (n = 3/time point). Blood was collected from the retro-orbital plexus throughout 336 h post dosing. The mean serum concentration–time profiles of HM15136 obtained after IV and SC administration to mice are shown in Fig. 4A. After single IV administration of HM15136 at a dose of 260 μg/kg in mice, AUC_last_ was 89,253.7 ng∙h/mL, C_0_ 5495.1 ng/mL,V_d_ 214.6 mL/kg, and CL was 2.7 mL/h/kg, indicating low clearance. After single SC administration of HM15136 at doses of 260 μg/kg, 519 μg/kg, and 1558 μg/kg in mice, AUC_last_ values were 68,605.9 ng∙h/mL, 268,111.5 ng∙h/mL, and 1,509,634.6 ng∙h/mL, respectively; C_max_ values were 1263.9 ng/mL, 4508.3 ng/mL, and 22091.1 ng/mL, respectively; T_max_ was 24.0 h for all groups; t_1/2_ values were 32.3 h, 56.2 h, and 53.8 h, respectively. SC bioavailability based on AUC_last_ was 76.9% in the 260 μg/kg dose group and more than 100% in the other groups. Power model analysis revealed that the increase in the AUC_last_ and C_max_ values of HM15136 over the range of 260 to 1558 μg/kg was greater than a dose-dependent increase (Table 1). These data from a mouse PK study indicate that HM15136 has a lower clearance rate than native glucagon.

**Figure 5:**
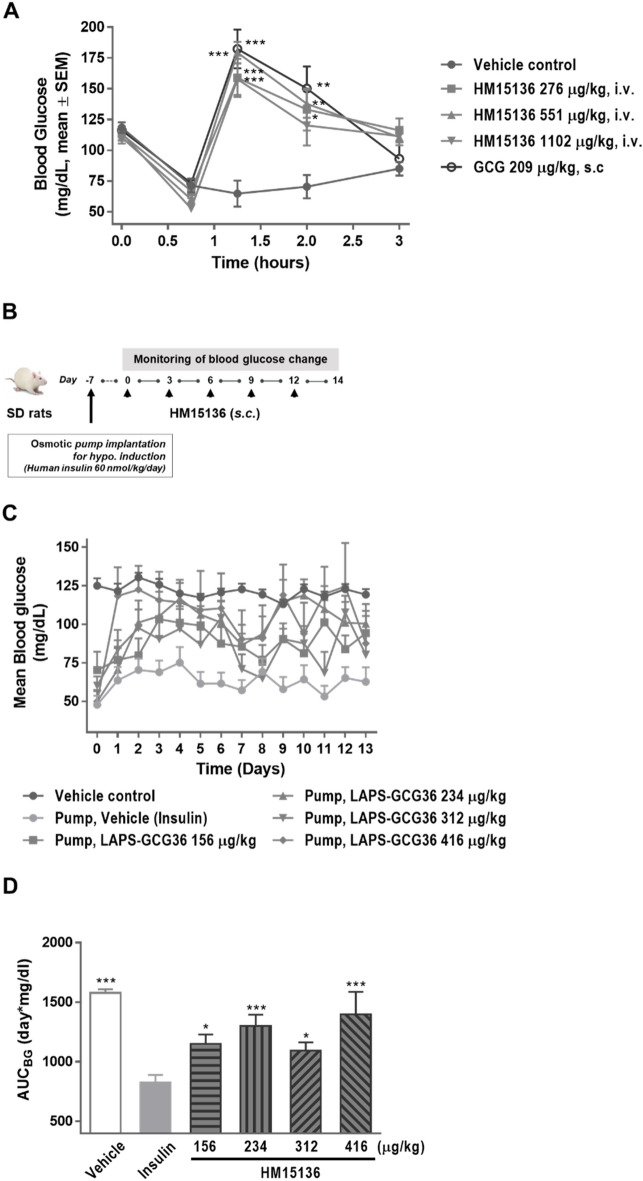
HM15136 increases blood glucose levels in hyperinsulinemia-induced acute and chronic hypoglycemic rats. (**A**) Hypoglycemia was induced with 0.65 IU/kg human insulin treatment in male SD rats fasted for 4 h. The indicated doses of HM15136 and native glucagon were administered intravenously once at 45 min (n = 5/group). Blood glucose of all animals were measured at 0, 45, 75, 120, and 180 min with OneTouch® Ultra® (Johnson & Johnson, USA). Each symbol represents the following: vehicle control, s.c. (filled circle), HM15136 268 μg/kg, i.v. (filled square), HM15136 536 μg/kg, i.v. (filled triangle), HM15136 1071 μg/kg, i.v. (filled reverted triangle), and glucagon 209 μg/kg, s.c. (open circle). (**B**) Experimental scheme for insulin-induced chronic hypoglycemia in SD rats. The indicated doses of HM15136 were subcutaneously administered every 3 days to mimic the human weekly dosing interval (n = 6–15/group). (**C**) Blood glucose levels with treatment over time. Each symbol represents the following; vehicle control for rats implanted with an insulin pump (filled circle), implanted pump containing vehicle in place of insulin (filled circle), HM15136 at 156 μg/kg (filled square), HM15136 at 234 μg/kg (filled triangle), HM15136 at 312 μg/kg (filled reverted triangle), and HM15136 at 416 μg/kg (filled diamond). (**D**) Quantification of the area under the curve (AUC) from blood glucose/time graph. Blood glucose after 2-h fasting was measured daily with OneTouch^®^ Ultra^®^ (Johnson & Johnson, USA). Each symbol represents the following; implanted pump containing vehicle in place of insulin (open square), vehicle control for rats implanted with an insulin pump (filled square), HM15136 at 156 μg/kg (square with horizontal stripes), HM15136 at 234 μg/kg (square with vertical stripes), HM15136 at 312 μg/kg (square with leftward cross stripes), and HM15136 at 416 μg/kg (square with rightward cross stripes). Values are expressed as mean ± SEM. (*p < 0.05 and ***p < 0.001 vs. Pump vehicle group).

Rat PK properties of HM15136 were evaluated after a single IV administration at a dose of 260 μg/kg and after SC administration at doses of 260 μg/kg, 519 μg/kg, and 1558 μg/kg in male SD rats (n = 3/group). Blood was collected from the jugular vein throughout 336 h post dosing. The mean (± S.D.) serum PK profiles are shown in Fig. 5B. After a single IV administration of HM15136 at a dose of 260 μg/kg in rats, AUC_last_ was 112,670.4 ± 22,228.3 ng∙h/mL, C_0_ 3757.0 ± 1196.0 ng/mL, V_d_ 207.1 ± 74.9 mL/kg, and the CL was 2.3 ± 0.5 mL/h/kg, indicating low clearance. After a single SC administration of HM15136 at doses of 260 μg/kg, 519 μg/kg, and 1558 μg/kg in rats, AUC_last_ values were 100,461.1 ± 12,112.2 ng∙h/mL, 307,765.0 ± 67,995.6 ng∙h/mL, and 1,842,306.4 ± 378,363.3 ng∙h/mL, respectively; C_max_ values were 905.7 ± 136.0 ng/mL, 2664.3 ± 362.6 ng/mL, and 18,938.4 ± 5627.8 ng/mL, respectively; T_max_ values were 48.0 ± 0.0 h, 64.0 ± 13.9 h, and 56.0 ± 13.9 h, respectively; and t_1/2_ values were 40.9 ± 17.7 h, 46.4 ± 1.8 h, and 54.8 ± 8.7 h, respectively. SC bioavailability based on AUC_last_ was 89.2% in the 260 μg/kg dose group and more than 100% in other groups. Similar to the results in mice, the increase in the AUC_last_ and C_max_ values of HM15136 was greater than a dose-dependent increase over the range of 260 to 1558 μg/kg, and there were significant differences in the dose-normalized AUC_last_ and C_max_ values between each group (p < 0.05, one-way ANOVA) (Table 1). Additionally, similar to the results obtained in mice, HM15136 showed a low clearance rate in rat and the drug lasts longer in serum at lower drug doses compared to the mouse PK profile.

Dog PK properties of HM15136 were evaluated after a single IV administration at a dose of 52 μg/kg and after SC doses of 52, 104, and 260 μg/kg administration in male beagle dogs (n = 3/group). Blood was collected from the cephalic vein throughout the 336 h post dosing. The mean (± S.D.) serum PK profiles are shown in Fig. 5C. After IV administration of HM15136 at a dose of 52 μg/kg, AUC_last_ was 22,309.9 ± 4093.9 ng∙h/mL; C_0_ 901.9 ± 72.1 ng/mL; V_d_ 77.3 ± 7.6 mL/kg; and CL was 2.2 ± 0.4 mL/h/kg, indicating low clearance. After SC administration of HM15136 at doses of 52 μg/kg, 104 μg/kg, and 260 μg/kg in dogs, AUC_last_ values were 27,271.2 ± 8067.6 ng∙h/mL, 81,676.7 ± 6672.7 ng∙h/mL, and 570,046.2 ± 65,377.0 ng∙h/mL, respectively; C_max_ values were 325.6 ± 104.0 ng/mL, 939.9 ± 186.5 ng/mL, and 4360.6 ± 612.4 ng/mL, respectively; T_max_ values were 32.0 ± 13.9 h, 56.0 ± 13.9 h, and 64.0 ± 13.9 h, respectively; and t_1/2_ values were 26.6 ± 1.5 h, 32.6 ± 12.9 h, and 34.9 ± 3.7 h, respectively. SC bioavailability based on AUC_last_ was more than 100% in all groups. The results of power model analysis revealed that the increase in the AUC_last_ and C_max_ values of HM15136 over the range of 52 to 260 μg/kg was greater than a dose-dependent increase, and there were significant differences in dose-normalized AUC_last_ and C_max_ values between each group (p < 0.05, one-way ANOVA) (Table 1). Pharmacokinetic parameters of dog for HM15136 were in line with those observed in mouse and rat, showing good bioavailability in all species examined.

To predict the human PK profile of HM15136, we used PK parameters from two doses (low and mid) in three animal species (mice, rats, and dogs) after SC administration. The human PK profiles were predicted under the assumption that HM15136 shows a linear PK profile in humans. The human CL/F ratio was predicted using simple allometry (SA) with the rule-of-exponent (ROE) method and the average of single-species scaling (SS_avg_) with a fixed exponent (0.8). The human V_SS_/F ratio was predicted using simple allometry (SA) and the average of single-species scaling (SS_avg_) with a fixed exponent (1.0). The predicted human CL/F values were 0.52 mL/h/kg (ROE) and 0.74 mL/h/kg (SS_avg_), and the predicted human V_SS_/F values were 102.2 mL/kg (SA) and 155.9 mL/kg (SS_avg_). There were some differences between the predicted values using different methods. Therefore, various simulation scenarios were applied using the combination of different CL/F and V_SS_/F values to predict human PK profiles (CL/F-V_SS_/F, scenario #1: ROE-SA; #2 : ROE-SS_avg_; #3 : SS_avg_-SA; #4 : SS_avg_–SS_avg_). Human PK profiles of HM15136 were predicted using the C_SS_-MRT method (PhoenixTM WinNonlin^®^ 8.0). The predicted human PK profiles are presented in Supplementary Fig. 2A–D, and the predicted human PK parameters are summarized in Supplementary Table 2. The predicted human AUC_0–28 day_ values of HM15136 after a single SC administration ranged from 71,820.8 ng∙h/mL to 102,572.2 ng∙h/mL (53 μg/kg) and 144,996.7 ng∙h/mL to 207,079.8 ng∙h/mL (107 μg/kg). The predicted half-lives ranged from 69.9 to 155.2 h. Following weekly dosing with HM15136, the predicted peak-to-trough ratio (PTR) ranged from 1.2 to 2.3, and the predicted accumulation ratios ranged from 1.4 to 3.0 for AUC_τ_ (area under the concentration–time curve during the dosing interval) and 1.4 to 2.4 for C_max_. In summary, the predicted human PK profiles of HM15136 had long-lasting properties with a long t_1/2_ (69.9–155.2 h) (Supplementary Table 2).

**Figure 6:**
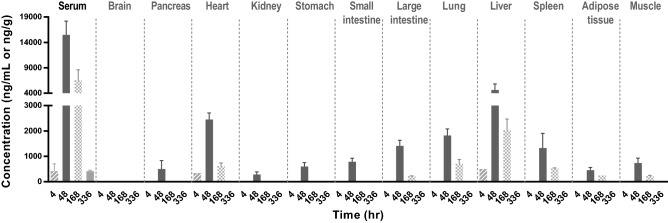
High levels of HM15136 were observed in the liver where gluconeogenesis and glycogenolyis occur.

Next, we examined tissue distribution of HM15136 after a single SC administration. We found the highest distribution of HM15136 in the liver. The mean (± S.D.) serum and tissue concentration–time profiles are presented in Fig. 6, and the tissue-to-serum ratio (T/S) values of each tissue are summarized in Table 2. At 4 h after SC administration of HM15136 at a dose of 1558 μg/kg in rats, the serum concentration of HM15136 was 416.0 ± 284.2 ng/mL. HM15136 was not detected in most tissues except the heart and liver in one of three rats. At 48 h after administration (serum max), the serum concentration of HM15136 was 15,500.8 ± 2686.9 ng/mL, and HM15136 was detected in the following tissues (listed in the order of decreasing concentration): liver, heart, lung, large intestine, spleen, small intestine, muscle, stomach, pancreas, adipose tissue, and kidney. T/S (tissue-to-serum) ratios in the liver, heart, lung, large intestine, spleen, small intestine, muscle, stomach, pancreas, adipose tissue, and kidney were 29.8%, 15.8%, 11.7%, 9.1%, 8.5%, 5.1%, 4.7%, 3.9%, 3.2%, 2.9%, and 1.8%, respectively. There was no distribution of HM15136 to the brain. At 168 h, the serum concentration of HM15136 was 6534.2 ± 2070.3 ng/mL, and the T/S ratios in the liver, lung, heart, spleen, large intestine, and muscle were 31.0%, 10.8%, 9.4%, 7.9%, 3.4%, and 3.4%, respectively. HM15136 was not detected in the brain, pancreas, kidney, stomach, small intestine, and adipose tissue. At 336 h, the serum concentration of HM15136 was 417.2 ± 36.1 ng/mL, but HM15136 was no longer present in any of the tissues examined (Table 2). These results suggest that HM15136 is highly distributed in the liver and detected in the liver even 7 days after administration of the drug.

**Table 2:** Tissue-to-serum ratio (%) and concentration of HM15136 after a single subcutaneous administration in rats.

Tissues	Concentration (ng/mL for serum or ng/g for tissue) and T/S ratio (%)							
	4 h		48 h		168 h		336 h	
	Conc (ng/mL, ng/g)	T/S (%)	Conc (ng/mL, ng/g)	T/S (%)	Conc (ng/mL, ng/g)	T/S (%)	Conc (ng/mL, ng/g)	T/S (%)
Serum	416.0 ± 284.2	–	15,500.8 ± 2686.9	–	6534.2 ± 2070.3	–	417.2 ± 36.1	–
Brain	NA	NA	NA	NA	NA	NA	NA	NA
Pancreas	NA	NA	499.8 ± 331.7	3.2	NA	NA	NA	NA
Heart	NA	NA	2447.4 ± 261.9	15.8	615.9 ± 124.3	9.4	NA	NA
Kidney	NA	NA	284.9 ± 105.5	1.8	NA	NA	NA	NA
Stomach	NA	NA	598.1 ± 152.9	3.9	NA	NA	NA	NA
Small intestine	NA	NA	785.5 ± 137.9	5.1	NA	NA	NA	NA
Large intestine	NA	NA	1411.9 ± 222.9	9.1	221.1 ± 11.0	3.4	NA	NA
Lung	NA	NA	1820.5 ± 258.7	11.7	703.1 ± 175.5	10.8	NA	NA
Liver	NA	NA	4625.4 ± 1216.9	29.8	2027.6 ± 444.5	31	NA	NA
Spleen	NA	NA	1323.7 ± 582.3	8.5	518.2 ± 29.5	7.9	NA	NA
Adipose tissue	NA	NA	453.9 ± 107.9	2.9	NA	NA	NA	NA
Muscle	NA	NA	735.4 ± 193.3	4.7	220.2 ± 30.3	3.4	NA	NA

*T/S ratio* tissue-to-serum ratio, *NA* not applicable.

### HM15136 administration raises blood glucose levels in acute and chronic hyperinsulinemia-induced hypoglycemic rat models

The BG-normalizing property of HM15136 was assessed in an insulin-induced acute hypoglycemia rat model^27^. As shown in Fig. 5A, single administration of HM15136 (276 μg/kg, 551 μg/kg, or 1102 μg/kg) induced a potent increase in BG levels (143.5%–176.9% vs vehicle control group) at 75 min (30 min after HM15136 treatment). Native glucagon also demonstrated similar efficacy (181.5% vs vehicle control group). Together with the ex vivo results in rat primary hepatocytes (Fig. 2B,C), these results suggest that HM15136 can exert the intrinsic activity of glucagon in vivo similar to native glucagon and thus increase BG levels in acute hypoglycemic rats.

An insulin-induced chronic hypoglycemic rat model was established to evaluate the therapeutic potential of HM15136 in hypoglycemic diseases. Briefly, SD rats were implanted with an osmotic pump filled with human insulin to continuously release insulin (60 nmol/kg/day) regardless of the BG level. One week following the pump implant, HM15136 was administered SC every 3 days at 156 μg/kg, 234 μg/kg, 312 μg/kg, and 416 μg/kg, and 2-h fasting BG levels were measured in each rat for 13 days (Fig. 5B). The data in Fig. 5C show that treatment with HM15136 produced a dose-dependent restoration of BG levels to near-normal levels (75–120 mg/dL) in chronically hypoglycemic rats. The area under the BG level × time curve (AUC_BG_) also demonstrated that HM15136 induced a dose-dependent increase in BG to near-normal levels (Fig. 5D). The administration of HM15136 at 156 μg/kg, 234 μg/kg, 312 μg/kg, and 416 μg/kg increased AUC_BG_ values to 1204.1 mg/dL∙day (p < 0.05), 1301.9 mg/dL∙day (p < 0.001), 1134.4 mg/dL∙day (p < 0.05), and 1398.4 (p < 0.001) mg/dL∙day, respectively, compared to AUC_BG_ levels from the insulin pump-implanted vehicle control rats (856.0 mg/dL∙day).

## Discussion

First discovered as a hyperglycemic factor, glucagon has been shown to play a critical role in maintaining glucose homeostasis by increasing glycogenolysis and gluconeogenesis. Glucagon has long been used in diabetic patients to treat acute severe hypoglycemia due to excess exogenous insulin^28^. Recently, its use has also been described in various chronic hypoglycemic diseases, such as CHI and hypoglycemia post-bariatric surgery^29^. CHI is caused by the elevation of blood insulin levels, which deplete BG. Herein, we have developed HM15136, a novel, long-acting glucagon analog, which could be applied for the treatment of CHI. HM15136 contains a novel glucagon analog (GC15136) conjugated to a recombinant human immunoglobulin G4 Fc fragment (HMC001) via a 10-kDa, bifunctional maleimide-polyethylene glycol-aldehyde (MAL-PEG-ALD) linker. HMC001 acts to prolong the duration of action of GC15136.

Patients with CHI have an abnormal insulin secretory capacity or an abnormal glucose-sensing mechanism, which results in the failure to reduce pancreatic insulin secretion even in the presence of hypoglycemia^6^. Approximately one third of children with CHI show abnormal neurodevelopment resulting in seizures, severe cognitive defects, and abnormal motor neuron development. Therefore, receiving proper treatment in a timely manner is crucial for preventing brain damage in patients^30^. Although glucagon is considered one of the most potent therapeutic options, its long-term utilization is limited due to poor solubility, limited stability at physiological pH, and short duration of action. Our results suggest that HM15136 increased intracellular cAMP levels in a dose-dependent manner, and that the maximal efficacy of HM15136 was similar to that of native glucagon, suggesting full agonist activity. Moreover, we confirmed that HM15136 promoted glucose production via both glycogenolysis and gluconeogenesis, similar to native glucagon, indicating that the pharmacological activity of HM15136 is analogous to that of native glucagon. The Fc moiety of HM15136 did not induce any immune-mediated effector activity. Our data from the in vivo efficacy study of HM15136 suggest that HM15136 not only rapidly reversed acute hypoglycemia but also normalized blood glucose levels in a rat model of chronic hypoglycemia. Notably, PK data reveal that HM15136 has high bioavailability and an extended half-life (t_1/2_) in mouse, rat, and dog models, consistent with recent clinical study data^31^. Further, HM15136 was mainly distributed to the liver, the target organ of glucagon for the induction of glycogenolysis and gluconeogenesis.

In the era of precision medicine, genetic and molecular diagnoses can be utilized for establishing suitable treatment strategies for each patient with CHI. Mutations in 14 genes regulating insulin secretion have been reported, among which, mutations in *ABCC8* and *KCNJ11* genes are the most common cause of CHI. Treatment strategies for CHI may depend on the histopathological and genetic etiology of the disease. Finding the optimal dosing regimen and the subpopulation of CHI patients who would be most benefited by the long-lasting glucagon analog is crucial for the successful development of HM15136.

Compared to other current soluble glucagon like Dasiglucagon (Zealand Pharma), HM15136 sustainably increases blood glucose levels in the CHI rat model. The half-life of HM15136 (T_1/2_ =  ~ 36 h) is at least 50 times longer than that of Dasiglucagon (T_1/2_ =  ~ 30 min). Although non-aqueous soluble glucagon (G-Pump, Xeris Pharmaceuticals) has high stability (up to 2 years in solution), it has been shown to induce more adverse effects such as injection site erythema, edema and discomfort.

Taken together, all these results in hypoglycemia rat models confirm the in vivo efficacy of HM15136 and support its continued development. HM15136 had higher solubility than native glucagon with an extended half-life and high bioavailability supported by in vivo data. Therefore, HM15136 could be a promising novel therapeutic agent and its long-acting properties could increase treatment adherence and improve the quality of life for patients with CHI.

## Materials and methods

### Materials

HM15136 and HMC001 were manufactured at Hanmi Bio Plant Complex (Pyeongtaek Gyeonggi-do, South Korea), and glucagon was purchased from Bachem (Switzerland). Identities of the peptides were verified by mass spectral analysis. Purity of the peptides was > 95% by HPLC analysis.

### Animals

All animal studies was approved by the Institutional Animal Care and Use Committee (IACUC) of the Hanmi Research Center, Republic of Korea. The IACUC and all procedures concerning the and use of laboratory animals were in accordance with joint guideline (11-1543061-000457-01) of Animal and Plant Quarantine Agency and Ministry of Food and Drug Safety, Republic of Korea. The study is reported in accordance with ARRIVE guidelines.

Sprague–Dawley rats were obtained from Orient Bio Inc. (Gyeonggi-do, South Korea) and given pellet chows and tap water ad libitum. Animals were randomly assigned to each group and housed on a 12-h light/12-h dark cycle at 22 ± 2 °C, 50 ± 20% relative humidity.

### cAMP assay

The cAMP level was investigated to determine the in vitro activity of HM15136 in CHO cells stably transfected with the human glucagon receptor, using a commercially available cAMP assay kit (PerkinElmer, USA).

### Culture of rat primary hepatocytes

Primary hepatocytes were isolated from an SD rat. Briefly, the liver was perfused with collagenase digestion medium, and the hepatocytes were collected using a 100 μm pore-size cell strainer and Percoll centrifugation. The isolated hepatocytes were plated in 24-well plates (2.5 × 10^6^ cells per well) in William’s complete medium and cultured at 37 °C and 5% CO_2_ in a humidified incubator.

### Glucose production assay

Glucose production in cultured rat primary hepatocytes was measured using a commercially available glucose assay kit (Sigma). Glucagon or HM15136 were serially diluted in DMEM containing gluconeogenic substrates (2 mM sodium pyruvate, 20 mM sodium lactate) for the evaluation of effects on gluconeogenesis and then added to rat primary hepatocytes for 6 h. In case of evaluation of effects on glycogenolysis, glucagon or HM15136 were serially diluted in DPBS and added to rat primary hepatocytes for 30 min. After 6 h or 30 min incubation, 100 μL of the sample was collected and mixed with 200 μL of the reagent in the glucose assay kit for 30 min. Next, 12 N H_2_SO_4_ was added, and the absorbance at 540 nm was measured. The glucose concentration was calculated using the optical density (OD) of the samples and standards.

### Binding affinity analysis between HM15136 and FcγRs

All experiments were carried out on a BIACORE T200 instrument (GE Healthcare, USA) at 25 °C. Amine coupling chemistry was used to immobilize FcγRs on the surface of a CM5 sensor chip. For activation, a mixture of NHS and EDC solution was injected onto a CM5 sensor chip for 7 min at a flow rate of 10 μL/min. FcγRIA, FcγRIIA, FcγRIIB/C, FcγRIIIA, and FcγRIIIB were immobilized on CM5 chips by injecting 5 μg/mL of FcγRIA, 10 μg/mL of FcγRIIA, 2 μg/mL of FcγRIIIA, 10 μg/mL of FcγRIIIB, and 10 μg/mL of FcγRIIB/C diluted with 10 mM sodium acetate (pH 4.5) onto CM5 chips at a flow rate of 5 μL/min. The target RU was 1500 RU for FcγRIA and FcγRIIA, 3,000 RU for FcγRIIB/C and FcγRIIIB, and 1,000 RU for FcγRIIIA. To deactivate unreacted moieties, the chip surface was blocked with 1 M ethanolamine/HCl (pH 8.5) for 7 min at a flow rate of 10 L/min. HMC001 and I.V.-Globulin SN were diluted with running buffer to 500, 250, 125, 62.5, and 31.25 nM. HM15136 was diluted with running buffer to 486, 243, 121.5, 60.75, and 30.38 nM. The test materials were injected for 10 min (for Fc immobilized with FcγRIA and FcγRIIA) or 15 min (for Fc immobilized with FcγRIIIA, FcγRIIIB, and FcγRIIB/C) for association, followed by 10 min-dissociation at 20 μL/min. Binding affinity was evaluated with a steady-state affinity fitting model in BIACORE T200 Evaluation Software version 3.0. Blank-subtracted sensograms (RU form reference channel and blanks were subtracted) were used for evaluation.

### Binding affinity analysis between HM15136 and C1q

I.V Globulin SN and HMC001 (20 μg/mL each) and 97.2 μg/mL of HM15136 diluted with 10 mM sodium acetate (pH 4.5) were injected onto a CM5 chip at a flow rate of 5 μL/min. The target immobilization of the three materials was 3000 RU. To stabilize the sensor chip, running buffer (HBS-EP) was injected for 10 min, followed by 10 min dissociation at 25 μL/min before test material injection. Then, regeneration buffer was injected for 30 s at 25 μL/min; this step was repeated three times. C1q was diluted with running buffer to 50, 25, 12.5, 6.25, and 3.125 nM and injected for 6 min for association, followed by 10-min dissociation at 25 μL/min. The remaining C1q was regenerated with regeneration buffer for 30 s at 25 μL/min; this step was repeated three times. Binding affinity was evaluated with a steady-state affinity fitting model in BIACORE T200 Evaluation Software version 3.0. Blank-subtracted sensograms (RU form reference channel and blank were subtracted) were used for evaluation.

### Measurement of blood glucose levels in an acute hypoglycemic rat model

After 4 h of fasting, all animals received insulin 0.65 IU/kg to induce hypoglycemia (40–80 mg/dL). All test agents (HM15136, glucagon) were administered 45 min after insulin administration. All animals had access to only water during the test. Fasting blood glucose was measured from the tail vein at 0 min (insulin 0.65 IU/kg injection point), 45 min (test agent time of injection), 75 min, 120 min, and 180 min using a OneTouch^®^ Ultra^®^ glucose meter (Johnson & Johnson, USA). The AUC data for the dose–response relationship of the glucose-increasing effects of the test agents were analyzed using GraphPad PRISM^®^, Version 6.07 (GraphPad Software, USA).

### Measurement of blood glucose levels in a chronic hypoglycemic rat model

After 2 h of fasting, HM15136 was administered at t = 0 h. All animals were allowed free access to water during the test. Fasting blood glucose was measured via the tail vein once daily using an OneTouch^®^ Ultra^®^ glucose meter (Johnson & Johnson, USA). The AUC data for the dose–response relationship was analyzed using GraphPad PRISM^®^, Version 6.07 (GraphPad Software, USA).

### Pharmacokinetic study

The general clinical signs were observed pre- and post dosing. Body weight was measured on the day of administration, and the dosing volume was set according to the body weight of each animal. To determine the effect of HM15136 administration on body weight changes, body weights of mice for 336 h sampling of each group were measured every day. Blood (0.3 mL) was collected from the retro-orbital plexus at 0.25, 0.5, 1, 4, 8, 24, 48, 72, 96, 120, 144, 168, 192, 216, 240, 264, 288, 312, and 336 h after IV administration and at 1, 4, 8, 24, 48, 72, 96, 120, 144, 168, 192, 216, 240, 264, 288, 312, and 336 h after SC administration of HM15136. Serum concentrations of HM15136 were determined using a modified ELISA assay. OD was determined at 450 nm using a microplate reader (Envision multilabel plate reader, PerkinElmer, USA). PK parameters of HM15136 were calculated from the average serum concentration–time data by a non-compartmental method using Phoenix™ WinNonlin^®^ 8.0 (Pharsight, USA). The peak serum concentration (C_max_) and the corresponding time (T_max_) were directly obtained from the raw data. The area under the serum concentration versus time curve (AUC_last_) was obtained by log-linear trapezoidal summation. Other PK parameters such as AUC from dosing time extrapolated to infinity (AUCINF), C_0_ (initial concentration), half-life (t_1/2_), volume of distribution (V_d_, V_d_/F), total body clearance (CL, CL/F), and mean residence time (MRT_last_) were calculated using WinNonlin. The absolute bioavailability (BA, %) of HM15136 was calculated using Eq. (1):

1 $${\text{BA }}\left( \% \right) \, = \, \left( {{\text{AUC s}}.{\text{c}}. \, /{\text{ AUC i}}.{\text{v}}.} \right) \, \times \, \left( {{\text{Dose i}}.{\text{v}}. \, /{\text{ Dose s}}.{\text{c}}.} \right) \, \times { 1}00.$$

### Statistical analysis

Statistical analysis was performed using the statistical program GraphPad PRISM®, Version 6.07 (GraphPad Software, USA). One-way ANOVA was used to evaluate significance. Multiple comparisons were performed with vehicle and positive control groups to determine whether there was a significant difference between the controls and test agent groups. A p value < 0.05 was considered statistically significant.

## Supplementary Information

Supplementary Information.
