# Supplementary material for: A novel glucagon analog with an extended half-life, HM15136, normalizes glucose levels in rodent models of congenital hyperinsulinism
Source: Sci Rep. 2022 Oct 6;12:16765. doi: 10.1038/s41598-022-21251-y (PMC9537296; doi:10.1038/s41598-022-21251-y)
Supplement: Supplementary file 1 — Supplementary Information. [file 41598_2022_21251_MOESM1_ESM.docx]

**Supplementary material**


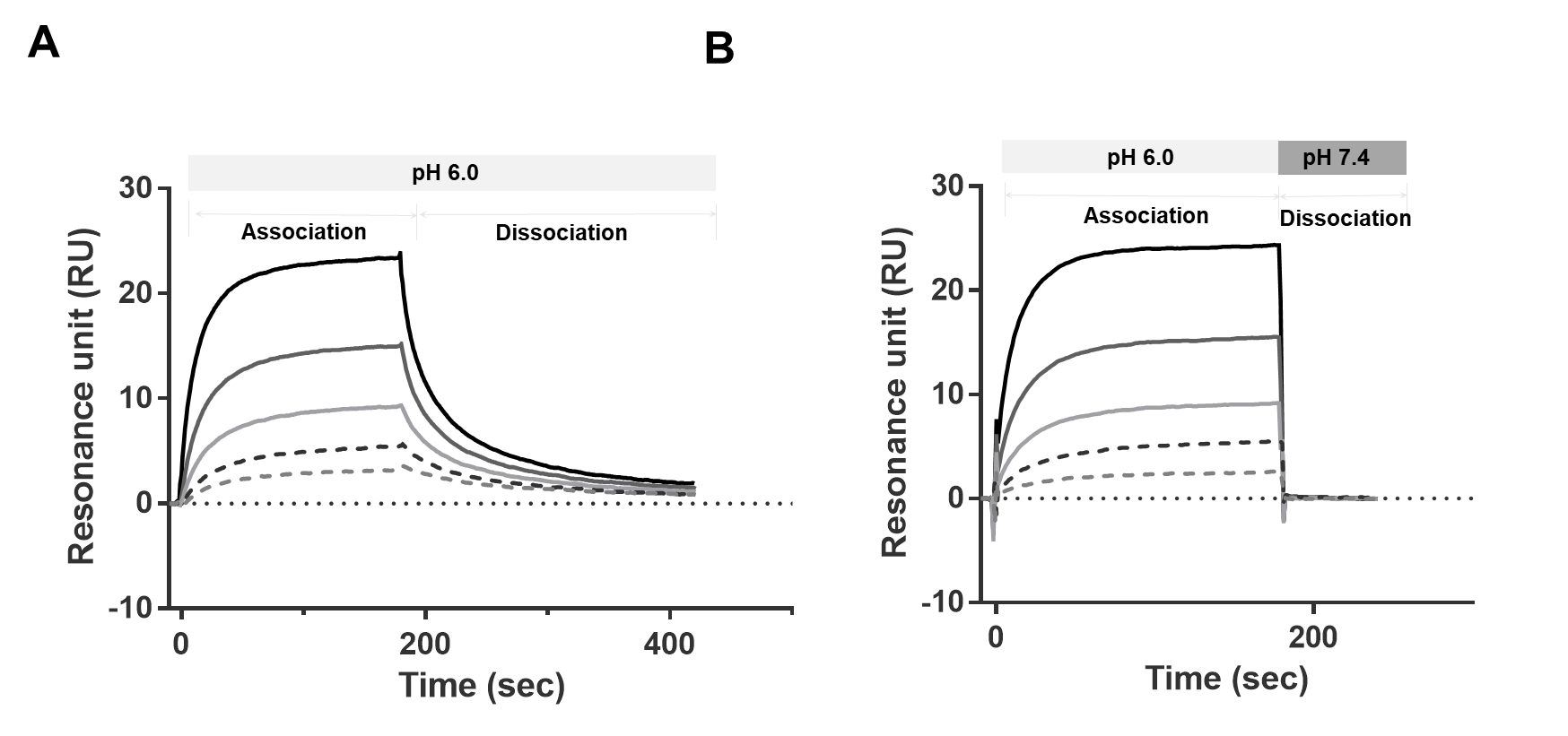


**Supplementary Fig. 1. HM15136 interacts with FcRn.** (A) Concentration-dependent binding of HM15136 to FcRn at acidic pH, (B) pH-dependent association and dissociation of HM15136 to FcRn upon switching from acidic to neutral pH. From the top to bottom, 97.2 nM, 48.6 nM, 24.3 nM, 12.2nM, and 6.1nM in both (A) and (B).


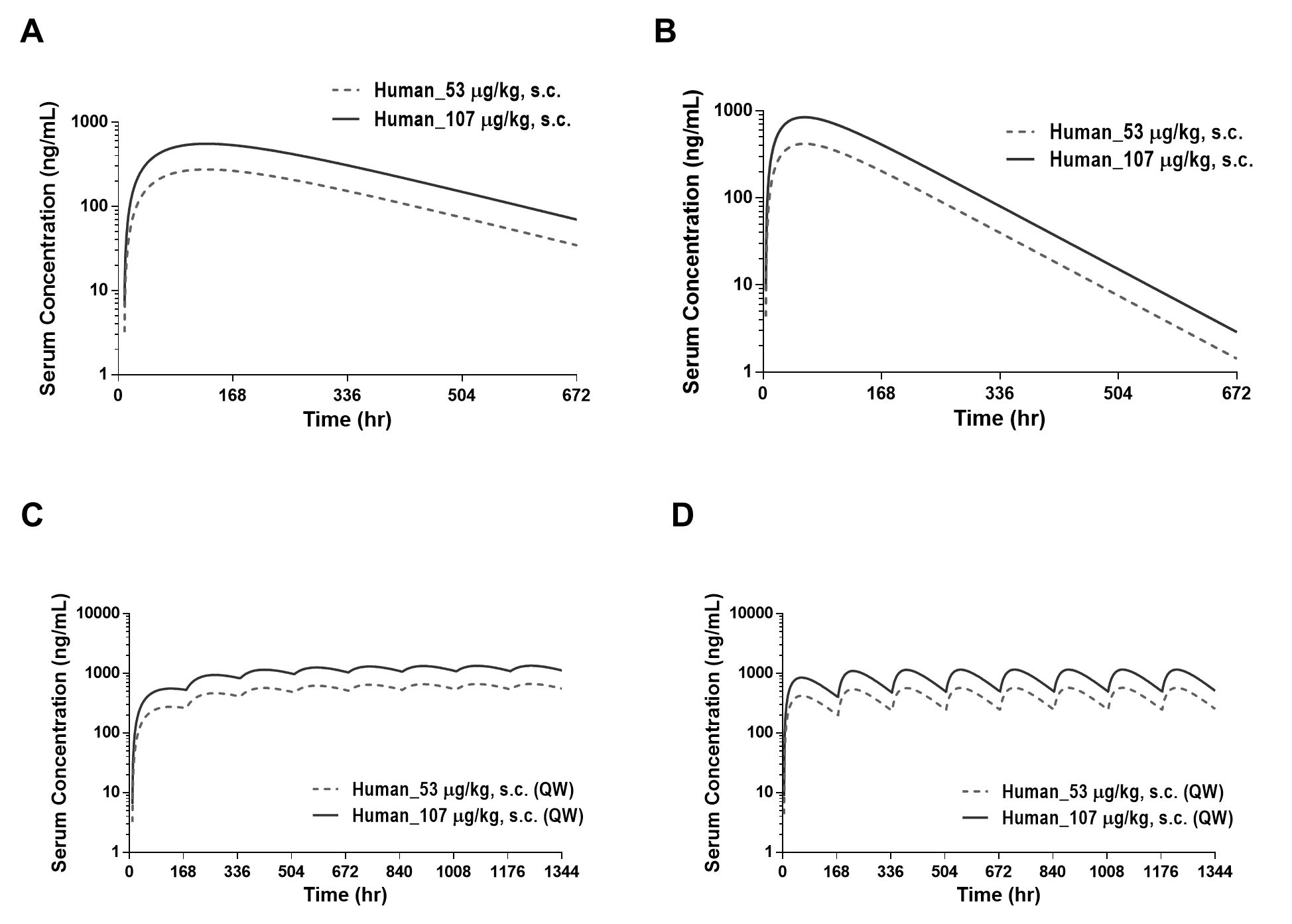


**Supplementary Fig. 2. HM15136 showed long-lasting predicted human PK profiles.** (A, B) Predicted serum concentration-time profiles of HM15136 after a single SC administration in human for the best case (A) and the worst case (B) The dashed and solid lines represent the 53 and 107 μg/kg doses, respectively. (C, D) Predicted serum concentration-time profiles of HM15136 after once-weekly SC administrations in humans for the best case (C) and the worst case (D). The dashed and solid lines represent the 53 and 107 μg/kg doses, respectively.

| **Test Materials** | **Binding affinities (K_D_, nM)** | | | | | |
| --- | --- | --- | --- | --- | --- | --- |
|  | **FcγRIA** | **FcγRIIA** | **FcγRIIB/C** | **FcγRIIIA** | **FcγRIIIB** | **C1q** |
| **IgG1**  **(=I.V.-Globulin SN)** | 26.7±2.6 | 184.0±29.1 | 333.9±19.0 | 209.2±9.8 | 1587.0±121.1 | 31.1±1.7 |
| **HMC001** | N.B. | N.B. | N.B. | N.B. | N.B. | N.B. |
| **HM15136** | N.B. | N.B. | N.B. | N.B. | N.B. | N.B. |

N.B.=No binding

**Supplementary Table 1**. Binding properties of HM15136 and HMC001 to FcγRs and C1q.

| **Test material** | **HM15136** | | | | | | | |
| --- | --- | --- | --- | --- | --- | --- | --- | --- |
| **Scenarios** | **#1 (ROE-SA)** | | **#2 (ROE-SS_avg_)**  **The best case** | | **# (SS_avg_-SA)**  **The worst case** | | **#4 (SS_avg_-SS_avg_)** | |
| **Dose (μg/kg)** | **53** | **107** | **53** | **107** | **53** | **107** | **53** | **107** |
| **Single-dose PK parameters** | | | | | | | | |
| C_max_ (ng/mL) | 418.5 | 844.9 | 275.1 | 555.3 | 420.0 | 847.9 | 276.4 | 558.0 |
| AUC_0-28day_ (ng·h/mL) | 102,572.2 | 207,079.8 | 96,858.4 | 1,955,44.4 | 728,17.6 | 147,009.2 | 71,820.8 | 144,996.7 |
| AUC_INF_ (ng·h/mL) | 104,104.9 | 210,174.1 | 104,595.6 | 211,164.7 | 72,961.6 | 147,299.7 | 73,243.5 | 147,869.0 |
| AUC_τ_ (ng·h/mL) | 55,490.6 | 112,028.2 | 34,642.0 | 69,937.5 | 51,905.9 | 104,791.2 | 36,668.3 | 74,028.4 |
| T_max_ (h) | 84.0 | | 129.0 | | 58.5 | | 90.0 | |
| t_1/2_ (h) | 100.4 | | 155.2 | | 69.9 | | 106.8 | |
| **Steady-state PK parameters (once weekly)** | | | | | | | | |
| C_max_ (ng/mL) | 720.0 | 1,453.7 | 664.0 | 1,340.5 | 571.8 | 1,154.5 | 498.3 | 1006.0 |
| C_trough_ (ng/mL) | 456.8 | 922.1 | 538.7 | 1,087.6 | 246.0 | 496.6 | 331.6 | 669.4 |
| AUC_τ_ (ng·h/mL) | 104,083.9 | 210,131.7 | 104,089.7 | 210,143.4 | 72,960.9 | 147,298.5 | 73,216.3 | 147,814.0 |
| PTR^(a)^ | 1.6 | | 1.2 | | 2.3 | | 1.5 | |
| Accumulation ratio | 1.9 (AUC) / 1.7 (C_max_) | | 3.0 (AUC) / 2.4 (C_max_) | | 1.4 (AUC) / 1.4 (C_max_) | | 2.0 (AUC) / 1.8 (C_max_) | |

^(a)^ PTR: peak-to-trough ratio

**Supplementary Table 2**. Predicted pharmacokinetic parameters of HM15136 after subcutaneous administration in humans.
